# Maternal and infant morbidity following birth before 27 weeks of gestation: a single centre study

**DOI:** 10.1038/s41598-020-79445-1

**Published:** 2021-01-11

**Authors:** Andrei S. Morgan, Saadia Waheed, Shivani Gajree, Neil Marlow, Anna L. David

**Affiliations:** 1 Research Department of Neonatology, Elizabeth Garrett Anderson Institute for Women’s Health, University College London, 2nd floor, Medical School Building, 74 Huntley Street, London, WC1E 6AU UK; 2 INSERM UMR 1153, Obstetrical, Perinatal and Pediatric Epidemiology Research Team (EPOPé), Centre for Epidemiology and Statistics Sorbonne Paris Cité, DHU Risks in Pregnancy, Paris Descartes University, Hôpital Tenon, Rue de la Chine, 75020 Paris, France; 3 SAMU 93-SMUR Pédiatrique, CHI André Gregoire, Groupe Hospitalier Universitaire Paris Seine-Saint-Denis, Assistance Publique des Hôpitaux de Paris, Montreuil, France; 4 Women’s Health Division, University College London Hospitals NHS Foundation Trust, 250 Euston Road, London, NW1 2PG UK; 5 NIHR University College London Hospitals BRC, Maple House, 149 Tottenham Court Road, London, W1T 7DN UK; 6 Research Department of Maternal Fetal Medicine, Institute for Women’s Health, University College London, 2nd floor, Medical School Building, 74 Huntley Street, London, WC1E 6AU UK

**Keywords:** Reproductive disorders, Pregnancy outcome, Reproductive signs and symptoms

## Abstract

Delivery at extreme preterm gestational ages (GA) $<\;27$ weeks is challenging with limited evidence often focused only on neonatal outcomes. We reviewed management and short term maternal, fetal and neonatal outcomes of births for 132 women (22 + 0 to 26 + 6 weeks’ GA) with a live fetus at admission to hospital and in labour or at planned emergency Caesarean section: 103 singleton and 29 (53 live fetuses) twin gestations. Thirty women (23%) had pre-existing medical problems, 110 (83%) had antenatal complications; only 17 (13%) women experienced neither. Major maternal labour and delivery complications affected 35 women (27%). 151 fetuses (97%) were exposed to antenatal steroids, 24 (15%) to tocolysis and 70 (45%) to magnesium sulphate. Delivery complications affected 11 fetuses, with 12 labour or delivery room deaths; survival to discharge was 75% (117/156), increasing with GA: 25% (1/4), 75% (18/24), 69% (29/42), 73% (33/45) and 88% (36/41) at 22, 23, 24, 25 and 26 weeks GA respectively (p = 0.024). No statistically important impact was seen from twin status, maternal illness or obstetric management. Even in a specialist perinatal unit antenatal and postnatal maternal complications are common in extreme preterm births, emphasising the need to include maternal as well as neonatal outcomes.

## Introduction

Delivery at extremely preterm gestional ages such as before 27 weeks of gestation poses major medical and ethical challenges. Despite increased numbers of extremely preterm babies born being admitted to intensive care in England in parallel with steady improvements in survival^1^, levels of short and long term infant morbidity remain high. Internationally, there is wide variation in guidance available to clinicians, much of which was developed without parental input^2^. Generally, most studies suggest that survival below 23 weeks of gestation is very unusual, although recent studies suggest that, where obstetric and neonatal management is oriented to optimising condition, survival at 22 and 23 weeks are similar and comprise 20-40% of live births receiving such “active care”^3^. Over 24 weeks of gestation full optimising care is often given. Parents need to receive accurate and targeted information from their expert multidisciplinary obstetric and neonatal team to allow them to reach consensus on the best way to provide care.

To reduce both maternal and neonatal morbidity associated with birth at extreme preterm gestational ages it is crucial that obstetric management is optimised but the evidence to support counselling is lacking^4^. Frequently at these extreme gestations, delivery is not expedited until a signal of maternal or fetal compromise is noted, so as to provide time for the fetus to gain days or weeks in utero. However, this comes at the expense of the mother who may herself subsequently experience major morbidity. Data on such maternal outcomes are lacking. Current evidence supports certain treatments—for example, use of antenatal steroids^5,6^—but is limited regarding interventions such as tocolysis^7,8^, magnesium sulphate prophylaxis^9^, augmentation of labour, fetal monitoring and episiotomy. There is even less information concerning the prevalence and management of complications during labour related to malpresentation^10,11^, cord prolapse^12^ and head entrapment^11,13^ or their effect on fetal and maternal outcomes. What is clear, however, is that delivery in a centre with a level 3 neonatal unit is associated with improved neonatal outcomes^14,15^; evidence suggests that survival is further enhanced in high throughput neonatal units^16,17^ and when there is a higher intensity of perinatal care^18,19^.

As the lead neonatal intensive care service of the North Central London Neonatal Network, University College London Hospitals NHS Foundation Trust (UCLH) admits a large number of women who deliver at extreme preterm gestational ages. In this study we carefully evaluated detailed data related to conduct of the labour and delivery of a large retrospective cohort of women at 22–26 completed weeks of gestation whose baby was alive at admission to UCLH and at the onset of labour or at the point of planned emergency Caesarean section. The objective was to perform a descriptive review of management and short term outcomes of deliveries and to quantify perinatal maternal and fetal complications when delivery occurs at extremely premature gestations. The aim is to aid clinicians in making management plans and counselling parents about likely outcomes.

## Methods

### Participants

This retrospective observational study was conducted over a three year period from 1st January 2011 to 31st December 2013 at University College London Hospital NHS Foundation Trust (UCLH). Women who delivered between 22^+0^ (22 weeks and 0 days) and 26^+6^ weeks of gestation were included if they had a live fetus at hospital admission and at the onset of labour, or at the point of planned emergency Caesarean section (for severe fetal growth restriction for example). We excluded women diagnosed with a late miscarriage or intrauterine death before labour onset or planned emergency Caesarean section. Cases were identified using the ECLIPSE maternity information system on which details of all mothers admitted to labour ward are recorded. This was cross-referenced with the hospital’s BadgerNet database on which standard information on all neonatal intensive care unit (NICU) admissions is entered. Obstetric data were collected from the hospital pathology and maternity ultrasound databases, and cross-referenced with maternal case notes. Neonatal data were obtained from BadgerNet and cross-referenced with discharge summaries. Ethical approval was waived as this study was deemed to be service evaluation and therefore not considered primary research. No members of the research team had access to identifiable patient data during the analysis.

### Outcomes

Both maternal and fetal/neonatal outcomes were considered. For mothers, we created two indicators of severe morbidity, for antenatal and labour/delivery complications, which included both life-threatening pathology and injuries threatening future pregnancies. For fetuses, we constructed an indicator of delivery complications which included unexpected intrapartum death, cord prolapse, head entrapment and unattended or traumatic delivery. We also report fetal survival to hospital discharge and selected neonatal morbidities for which data were available and considered reliable among surviving babies.

### Variables collected

Antenatal variables collected included maternal age, ethnicity (categorised as white, black, Asian (including Indian, Pakastani and Bangladeshi) and mixed/other origin), pre-existing maternal disease, obstetric complications (antepartum haemorrhage, pre-eclampsia and related syndromes, gestational hypertension, preterm prelabour rupture of membranes (PPROM) and sepsis), and management (cervical cerclage, antenatal transfer, fetal monitoring in labour, use of tocolysis, steroids, and magnesium sulphate). We created an overall indicator for antenatal pregnancy complications comprising the above obstetric complications plus cervical cerclage, and antenatally diagnosed fetal complications (fetal growth restriction or abnormal amniotic fluid volume). Intrapartum variables collected included gestational age at delivery (in completed weeks of gestation), labour onset (spontaneous, induced or none), sepsis and haemorrhage, fetal presentation and monitoring in labour, mode of delivery and birth difficulties, birth weight (categorised as < 500, 500–649, 650–799 and 800+ g), and fetal sex. Neonatal variables included admission to neonatal unit, Apgar score and use of surfactant. We also collected information on the seniority of the persons conducting the delivery and leading resuscitation of the baby.

### Statistical analysis

We performed a descriptive analysis taking into consideration different baseline populations using cross-tabulations and assessing means and proportions. As fetuses in multiple pregnancies may not be delivered at the same time (i.e. there may be a delay of one or more days between delivery of one fetus and the next), we report results on both a per-mother and per-fetus (or per-neonate) basis, as appropriate. Statistical significance was defined a priori as a two-sided p-value $<0.05$ and was assessed using chi-squared tests or logistic regression if there were more than two categories. All analyses were performed using R version 3.3.3 (“Another Canoe”)^20^. Reporting was carried out in accordance with the STROBE guidelines^21^ (see Supplementary Note).

**Figure 1:**
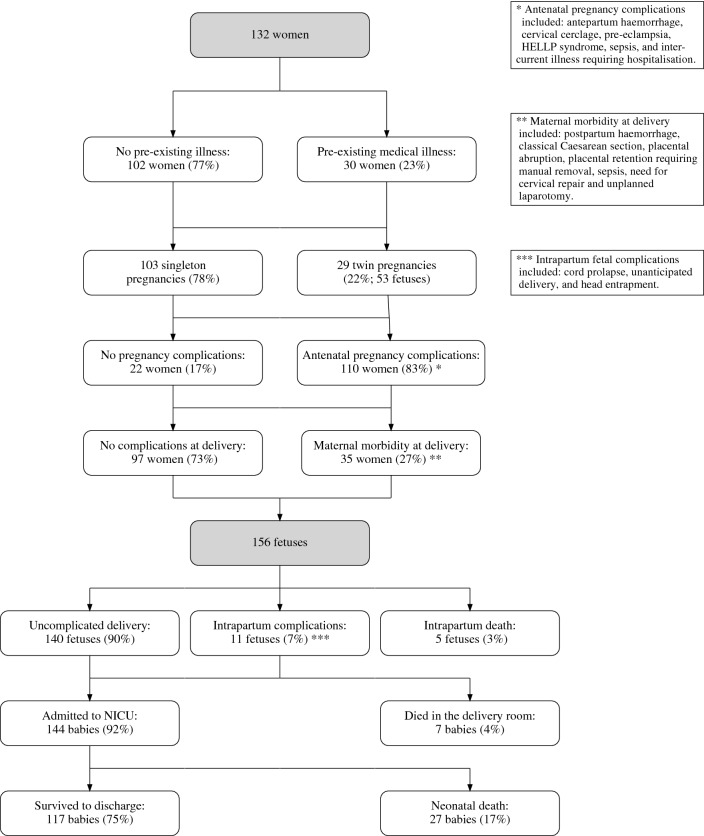
Flow chart of women and fetuses/babies (alive at the onset of labour or when a decision was made to perform Caesarean section) delivered at 22 + 0 to 26 + 6 completed weeks of gestation at University College London Hospitals NHS Foundation Trust between 1st January 2011 and 31st December 2013.

**Table 1:** Information for fetuses (or babies) born between 22 + 0 and 26 + 6 completed weeks of gestation between 1st January 2011 and 31st December 2013 at University College London Hospital NHS Foundation Trust who were alive at the onset of labour or when a decision was made to perform Caesarean section.

Gestational age (weeks)	22	23	24	25	26	22–26
N (%)	4 (2.6)	24 (15.4)	42 (26.9)	45 (28.8)	41 (26.3)	156 (100.0)
**Pregnancy type**						
Singleton	1	15	26	34	27	103
Multiple	3	9	16	11	14	53
**Exposure to antenatal treatments**						
Tocolysis	2	3	10	6	3	24
Antenatal steroids	3	24	42	43	39	151
Magnesium sulphate	2	0	26	18	24	70
**Labour monitoring**						
CEFM	0	2	4	15	20	41
Intermittent auscultation	4	20	36	29	16	105
None	0	2	2	1	5	10
**Mode of delivery**						
Vaginal	4	21	34	36	21	116
Caesarean	0	3	8	9	20	40
**Presentation at delivery**						
Cephalic	2	15	19	21	24	81
Breech	2	8	19	21	14	64
Transverse	0	1	4	3	3	11
**Obstetrician at delivery**						
Consultant	0	6	20	15	23	64
Registrar	2	11	18	24	17	72
Midwife	1	5	3	4	1	14
None/unknown	1	2	1	2	0	6
**Neonatologist at delivery**						
Consultant	4	16	22	27	23	92
Registrar	0	5	16	13	17	51
None/unknown	0	3	4	5	1	13
**Sex**						
Female	2	9	20	24	14	69
Male	2	15	22	21	27	87
**Birth weight (g)**						
$<500$	2	4	0	1	0	7
500–649	2	17	18	10	4	51
650–799	0	3	21	21	13	58
800+	0	0	3	13	24	40
**Survival status**						
Perinatal death	1	2	4	4	1	12
Died on NICU	2	4	9	8	4	27
Survived to discharge	1	18	29	33	36	117
**Morbidity among survivors to discharge**						
No IVH	1	13	21	25	29	89
IVH grade 1 or 2	0	3	3	5	4	15
IVH grade 3 or 4	0	2	5	3	3	13
NEC	0	2	1	2	3	8

*CEFM* continuous external fetal monitoring, *NICU* neonatal intensive care unit, *IVH* intraventricular haemorrhage, graded according to Papille’s classification, *NEC* necrotising enterocolitis, Bell’s classification stage 2 or 3).

## Results

### Baseline population

Between 1st January 2011 and 31st December 2013, 132 women delivered 156 babies between 22^+0^ and 26^+6^ weeks of gestation; all had one or more fetuses alive at the onset of labour (or when a decision was made to perform planned emergency Caesarean section). Ninety one (69%) women underwent in utero transfer from another hospital. Complete data were available for maternal and fetal outcomes in all cases; not all women had an ultrasound at UCLH after in utero transfer. For 117 women, ethnicity data were available: 51 (44%) women were described as white, 35 (30%) as black, 26 (22%) as Asian and five (4%) as mixed or other origin.

Of the 132 women, 103 (78%) had singleton pregnancies; 29 twin pregnancies accounted for the other 53 fetuses, including five with only one baby alive at the start of labour (of their respective twins, one had severe fetal growth restriction, one died at home prior to admission and another in hospital prior to the onset of labour, one was a co-existent hydatidiform mole, and one was delivered outside the study period). There were no higher order multiple pregnancies delivered during the study period. No mothers died but 35 women (27%) experienced severe morbidity associated with delivery (see Fig. 1). Five fetuses died during labour and a further seven died in the delivery room; overall, 117 babies (75%) survived to hospital discharge. Complications and morbidities are shown in the Fig. 1 and broken down by gestational age in the Table 1.

### Maternal health factors

Mothers ranged in age from 17 to 53 (median age 32, interquartile range 28–35). Thirty mothers (23%) had pre-existing health problems, including type 1 or 2 diabetes (n = 5), thyroid disease (n = 5), and essential hypertension or renal disease (n = 5). Mothers also experienced asthma (n = 4), uterine anomalies (n = 3), as well as other autoimmune diseases, gastrointestinal surgical conditions, cardiac disease, blood disorders, substance misuse, depression, psychosis, hepatitis (B and C), and cancer; some mothers had more than one condition. Six women received donor egg in vitro fertilisation due to female factor infertility, of which three pregnancies underwent fetal reduction (two from quadruplets and one from triplets) down to twin pregnancies.

### Antenatal management and complications

During their pregnancy, 110 women (83%, with 129 fetuses (83%)) experienced new onset pathology. These included 25 (19%) women who experienced antepartum haemorrhage (APH), defined as requiring hospital admission with iron therapy and/or blood transfusion, 21 women (16%) who had a short cervical length and received cervical cerclage, eight women (6%) diagnosed with pre-eclampsia and two (2%) with HELLP (haemolysis, elevated liver enzymes and low platelets) syndrome. Other diagnoses included gestational diabetes requiring insulin, acute appendicitis during the first trimester which was managed conservatively, ascites associated with severe pre-eclampsia, as well as hydatidiform mole and intrauterine fetal death of a co-twin. Conditions are not exclusive, and there was no association with ethnicity (p> 0.99).

In the 117 women (136 fetuses) for whom a UCLH ultrasound scan examination was performed, 14 fetuses (10%) had anhydramnios and 25 (18%) oligohydramnios; one had polyhydramnios. Twenty one fetuses (15%) were diagnosed with fetal growth restriction, but only one had an estimated fetal weight below 500 g.

Nineteen women (14%) received tocolysis, the most common indications being during in utero transfer to UCLH (15 women) from referring hospitals and/or alongside antenatal steroid administration. Overall, 127 women (96%) received antenatal steroids, and only five fetuses (3%) had no exposure to steroids; just one of these, weighing less than 500 g, did not survive. Magnesium sulphate was administered to 55 mothers [42%, 70 fetuses (45%)]; it was not used for 43 fetuses (28%) because of rapid delivery (39 fetuses) or cord prolapse and emergency delivery (4 fetuses), and not considered for 21 fetuses (13%). For the remaining 16 fetuses, magnesium sulphate was not prescribed due to contraindications: APH (7), maternal sepsis (2) or because the gestational age was below 24 weeks (7).

### Labour and delivery management and complications

Sixty-seven women (51%, 77 fetuses (49%) had preterm premature rupture of membranes, and the majority of women (104, 79%) had spontaneous preterm labour; six women were induced due to maternal sepsis, and 21 had Caesarean section prior to labour (indications included one or more of the following: severe pre-eclampsia, major antepartum haemorrhage, maternal sepsis, severe fetal growth restriction, previous classical Caesarean section). Monitoring in labour varied according to gestational age and is described in the Table 1. Ultrasound assessment was used during the labour of 79 (51%) fetuses. In each case, fetal presentation was confirmed; ultrasound was also used to confirm viability during labour (n = 70) and to assess labour progression (n = 17). Artificial rupture of membranes was used to induce or augment labour for 10 fetuses (6%) and intravenous oxytocin augmentation for 27 (17%): 14 during the first stage and 13 during the second stage.

Ultimately, 116 (74%) fetuses were delivered vaginally and 40 (26%) by Caesarean section. Just over a quarter of women (34, 26%) underwent Caesarean delivery, including one who delivered her first twin fetus vaginally. Fetal presentation in almost half (75 fetuses, 48%) was non-cephalic: 64 (41%) were breech and 11 (7%) were transverse.

### Maternal outcomes

Thirty-five women (27%) had a maternal complication relating to delivery; the most common being postpartum haemorrhage (13 women, 10%), sepsis (11 women, 8%), placental abruption (8 women, 6%), retained placenta requiring manual removal (7 women, 5%), unplanned laparotomy (3 women), and need for cervical repair (2 women); some women had more than one complication, and three (2%) required admission to the intensive care unit. There was no association between pre-existing medical disease and complications during pregnancy (p> 0.99), between pre-existing medical disease or complications during pregnancy with the occurrence of maternal morbidity at the time of delivery (p> 0.99 and 0.20, respectively), or between maternal ethnicity and maternal morbidity at the time of delivery (p > 0.99).

### Fetal/baby outcomes

Only one of the five intrapartum deaths was unexpected; the other four were associated with prolonged rupture of membranes and anhydramnios in two cases, and extreme fetal growth restriction and a gestational age of 22 weeks in the other two, with a decision made in each case not to provide active support. A further 11 babies (7%) experienced complications during delivery. These included cord prolapse (4), unanticipated rapid delivery (3 babies: in the bathroom, in the antenatal ward, and one while the mother was unattended in the delivery room) and head entrapment (3) as well as one case of trauma at delivery due to rapid delivery of the baby in the membranes and a subsequent fall to the floor. Seven babies died without admission to neonatal intensive care: resuscitation was attempted in only one of these cases, following precipitate vaginal delivery accompanied by placental abruption. Overall, 139 fetuses (89%) received surfactant in the delivery room and 144 (92%) were admitted to NICU. Apgar scores were available for 137 of the 151 live births at 1 minute, with an overall mean of 3.8 [standard deviation (SD) 2.4]; at 22, 23, 24, 25 and 26 weeks, the means (SDs) were 1.0 (1.0), 2.8 (2.3), 3.5 (2.2), 3.9 (2.6) and 4.7 (2.2) respectively. For the 134 babies with data at 5 minutes, the overall mean was 6.6 (2.6) with mean (SD) of 3.7 (4.0), 5.4 (3.1), 6.3 (2.5), 6.7 (2.6) and 7.5 (1.8) at 22, 23, 24, 25 and 26 weeks, respectively.

Fetal survival to hospital discharge was associated with increased gestational age at delivery (p = 0.024), but was not related to fetal presentation, delivery mode, other management or maternal ethnicity. Specifically, antenatal magnesium sulphate was not associated with survival, nor with head scan status among those born alive or who survived to discharge. In the group with antenatal ultrasound data available, survival was related to the amount of amniotic fluid present: only 24 of 38 (63%) fetuses with oligo or anhydramnios survived compared with 76 of 97 (78%) with normal or increased amniotic fluid volume (p = 0.04). There was no association between antenatal diagnosis of fetal growth restriction and fetal survival (p = 0.30). No factors were identified as being predictive of fetal complications at delivery, nor was there any association seen between fetal or neonatal complications and maternal outcomes.

## Discussion

This study demonstrates the high prevalence of perinatal complications for both mothers and fetuses delivering between 22 and 26 completed weeks of gestation, despite management in a regional tertiary referral centre with a high throughput of these pregnancies. More than one in five women had a pre-existing medical condition, more than four in five experienced new onset pathology during the pregnancy, and over a quarter of mothers had a complication relating to delivery. Despite almost 80% of women having spontaneous labour, over a quarter were delivered by Caesarean section, with an important number of women requiring other intervention in theatre (laparotomy, cervical repair, manual removal of placenta). However, more than nine in ten fetuses survived labour, the majority were exposed to antenatal steroids and given surfactant during initial stabilisation, and three-quarters survived to hospital discharge.

The strength of this study is that it was conducted at a single unit with extremely focussed data collection that garnered obstetric information from labour and delivery records, rather than just at admission to NICU. This permitted detailed elaboration of circumstances relating to maternal and fetal complications at delivery. We included all mothers and fetuses that were alive at admission to hospital and the beginning of labour or the decision for a planned emergency Caesarean section which is important as these are the pregnancies where timely obstetric intervention may affect outcome. Further strengths are that UCLH, as a regional referral centre within a densely populated urban environment, cares for a large number of women with threatened extremely preterm labour and has developed protocols specifically relating to the active management of these deliveries. For example, guidelines for the use of magnesium sulphate for cerebral palsy prophylaxis were introduced in 2010 for all women delivering below 30 weeks of gestation—ahead of similar guidance from the Royal College of Obstetricians and Gynaecologists^22^. Still, not all women received magnesium sulphate during delivery. This was primarily because of rapid delivery or contraindication, but in an important of number of cases it was not considered at that time.

In contrast, that this is a single centre study is also a limitation. We highlight that deliveries at extremely preterm gestations have very high rates of complications both for the mother and the fetus, but are unable to adequately quantify these in a manner that can be extrapolated to other populations. This is because the subjects admitted to our service do not come from a strictly defined population, although a majority are from our defined geographic network population, and therefore we were unable to take into consideration broader sociodemographic factors. We wanted to investigate the effects of interventions, but overall numbers were small and homogeneity of management (for example, the almost uniform administration of antenatal steroids) did not allow for differences between groups of patients to be easily identified.

Despite the high proportion of mothers in spontaneous labour, a large number were delivered by Caesarean delivery. This may prevent some of the unpredictable complications of a labour but must be balanced against the added morbidity to the mother. Current evidence suggests that delivery method should be based on obstetric or maternal indications rather than perceived outcome for the baby and Caesarean delivery cannot be recommended routinely^6,23–26^, although it may reduce the occurrence of intraventricular haemorrhage^27^. A pilot feasibility trial (the CASSAVA study) to address the question of mode of delivery at extreme preterm gestations has been funded in the UK (NIHR 17/22 Mode of delivery for preterm infants).

More than two-thirds of women who were delivered in this cohort were transferred antenatally from other network hospitals in our area, reflecting current policy. Over 90% of fetuses in our cohort were admitted to NICU, and three quarters of the total population survived to discharge. This is in keeping with results from Sweden^28,29^ and Japan^30,31^ although of course our numbers are smaller and confidence intervals therefore wider. In England, the EPICure studies demonstrated increased admissions to NICU between 1995 and 2006^1^, probably linked to an increase in the numbers of babies born at these gestations as well as to changes in attitudes in the delivery room^32^. Moreover, EPICure 2 showed that higher volume neonatal units have better outcomes for babies^16^, a finding replicated in other studies^17,33^.

There is a paucity of literature looking specifically at delivery complications following extremely preterm delivery. A single centre study from New Zealand studied babies born at 23 and 24 weeks’ gestation over a 10 year period and demonstrated rates of survival without moderate or severe disability at two years of age of 58% and 74%, respectively^34^. However, they focussed exclusively on neonatal outcomes and provided much less detail about perinatal circumstances. A Canadian Perinatal Network study did focus on maternal outcomes, but included only those who delivered at 23 completed weeks’ gestation^35^. Our study is unusual as it focuses primarily on obstetric management and very short term outcomes for all deliveries between 22 and 26 completed weeks of gestation.

This study demonstrates that delivery at extremely preterm gestations, even in an urban centre with a high number of deliveries at these gestations, is associated with a high rate of complications to mothers and babies. The findings support the notion that women with threatened extremely preterm delivery should be transferred at the earliest possibility to a center with sufficient expertise to manage both the mother and fetus during labour as well as neonatal expertise for the newborn baby. The data highlight the importance of a combined approach to extremely premature delivery by obstetricians and neonatologists to facilitate parental involvement in decision-making, and demonstrate why it is important to include maternal as well as neonatal outcomes when examining outcomes of extremely preterm birth.

## Supplementary Information

Supplementary Information.

## Data Availability

The data analysed during the current study are not publicly available due to the potential for re-identification of individual participants; however, they are available from the corresponding author on reasonable request to investigators who meet the criteria for access to confidential patient data.
