# Supplementary material for: Maternal and infant morbidity following birth before 27 weeks of gestation: a single centre study
Source: Sci Rep. 2021 Jan 11;11:288. doi: 10.1038/s41598-020-79445-1 (PMC7801674; doi:10.1038/s41598-020-79445-1)
Supplement: Supplementary file 1 — Supplementary Information. [file 41598_2020_79445_MOESM1_ESM.pdf]

Supplementary Note: STROBE Checklist for ‘Maternal and infant morbidity following birth before 27 weeks of gestation: a single centre study”

Andrei S. Morgan[1,2,3,4,+], **Saadia Waheed**[4,+], **Shivani Gajree**[4,], **Neil Marlow**[1,4,5], **and Anna L. David**[4,5,6,*]

[1]Research Department of Neonatology, Elizabeth Garrett Anderson Institute for Women’s Health, University College London, 2nd floor, Medical School Building, 74 Huntley Street, London WC1E 6AU
[2]INSERM UMR 1153, Obstetrical, Perinatal and Pediatric Epidemiology Research Team (EPOPé), Centre for Epidemiology and Statistics Sorbonne Paris Cité, DHU Risks in Pregnancy, Paris Descartes University, Hôpital Tenon, Rue de la Chine, 75020 Paris, France.
[3]SAMU 93 - SMUR Pédiatrique, CHI André Gregoire, Groupe Hospitalier Universitaire Paris Seine-Saint-Denis, Assistance Publique des Hôpitaux de Paris, Montreuil, France.
[4]Women’s Health Division, University College London Hospitals NHS Foundation Trust, 250 Euston Road, London NW1 2PG, UK
[5]NIHR University College London Hospitals BRC, Maple House, 149 Tottenham Court Road, London W1T 7DN
[6]Research Department of Maternal Fetal Medicine, Institute for Women’s Health, University College London, 2nd floor, Medical School Building, 74 Huntley Street, London WC1E 6AU
*a.david@ucl.ac.uk
+these authors contributed equally to this work

ABSTRACT

STROBE checklist for “Maternal and infant morbidity following birth before 27 weeks of gestation: a single centre study”

Description

The STrengthening the Reporting of OBservational studies in Epidemiology (STROBE) checklist contains a list of items to be included in reports of observational studies.[1] The following table contains the STROBE checklist for the article, “*Maternal and infant morbidity following birth before 27 weeks of gestation: a single centre study*” alongside a reference to where in the article the information may be found.

STROBE checklist

	Item No	Recommendation	Section (notes)
**Title and abstract**	1	(a) Indicate the study’s design with a commonly used term in the title or the abstract	Title (“a single centre study”)
		(b) Provide in the abstract an informative and balanced summary of what was done and what was found	Abstract: “We reviewed management and short term maternal, fetal and neonatal outcomes of births for 132 women (22+0 to 26+6 weeks’ GA) with a live fetus at admission to hospital and in labour or at planned emergency Caesarean section”

Continued on next page…

Strobe checklist (continued)

	Item No	Recommendation	Section (notes)
**Introduction**			
Background/ rationale	2	Explain the scientific background and rationale for the investigation being reported	Introduction (paragraphs 1 to 3)
Objectives	3	State specific objectives, including any prespecified hypotheses	Introduction (final paragraph)
**Methods**			
Study design	4	Present key elements of study design early in the paper	Methods section
Setting	5	Describe the setting, locations, and relevant dates, including periods of recruitment, exposure, follow-up, and data collection	Methods ("Participants" section)
Participants	6	(a) Give the eligibility criteria, and the sources and methods of selection of participants. Describe methods of follow-up	Methods ("Participants" section)
		(b) For matched studies, give matching criteria and number of exposed and unexposed	N/A
Variables	7	Clearly define all outcomes, exposures, predictors, potential confounders, and effect modifiers. Give diagnostic criteria, if applicable	Methods (sections on "Outcomes" and "Variables collected".
Data sources/ measurement	8	For each variable of interest, give sources of data and details of methods of assessment (measurement). Describe comparability of assessment methods if there is more than one group.	Methods (sections on "Variables collected" and "Statistical analysis".
Bias	9	Describe any efforts to address potential sources of bias	Methods ("Statistical methods" section.)
Study size	10	Explain how the study size was arrived at	Methods ("Study population") and Figure.)
Quantitative variables	11	Explain how quantitative variables were handled in the analyses. If applicable, describe which groupings were chosen and why	Methods ("Variables collected")
Statistical methods	12	(a) Describe all statistical methods, including those used to control for confounding	Methods (sections on "Statistical methods")
		(b) Describe any methods used to examine subgroups and interactions	Methods (Statistical methods)

Continued on next page...

Strobe checklist (continued)

	Item No	Recommendation	Section (notes)
		(c) Explain how missing data were addressed	This was a descriptive analysis of data collected from a single hospital; there were no missing data other than for antenatal ultrasound, as described in the first paragraph of the results.
		(d) If applicable, explain how loss to follow-up was addressed	N/A
		(e) Describe any sensitivity analyses	This was a descriptive study and hence no sensitivity analyses were performed.
**Results**			
Participants	13	(a) Report numbers of individuals at each stage of study—eg numbers potentially eligible, examined for eligibility, confirmed eligible, included in the study, completing follow-up, and analysed	Results (paragraphs 1 and 2) and Figure.
		(b) Give reasons for non-participation at each stage	N/A
		(c) Consider use of a flow diagram	Figure.
Descriptive data	14	(a) Give characteristics of study participants (eg demographic, clinical, social) and information on exposures and potential confounders	Results and Table.
		(b) Indicate number of participants with missing data for each variable of interest	There were no missing data for the main variables.
		(c) Summarise follow-up time (eg, average and total amount)	N/A
Outcome data	15	Report numbers of outcome events or summary measures over time	Results and Table.
Main results	16	(a) Give unadjusted estimates and, if applicable, confounder-adjusted estimates and their precision (eg, 95% confidence interval). Make clear which confounders were adjusted for and why they were included	This is a descriptive study and there are no estimates. We included the means and standard deviations of Apgar scores at 1 and 5 minutes of age.
		(b) Report category boundaries when continuous variables were categorized	Table.

Continued on next page…

Strobe checklist (continued)

	Item No	Recommendation	Section (notes)
		(c) If relevant, consider translating estimates of relative risk into absolute risk for a meaningful time period	N/A
Other analyses	17	Report other analyses done — eg analyses of subgroups and interactions, and sensitivity analyses	N/A
**Discussion**			
Key results	18	Summarise key results with reference to study objectives	Discussion (paragraph 1)
Limitations	19	Discuss limitations of the study, taking into account sources of potential bias or imprecision. Discuss both direction and magnitude of any potential bias	Discussion (paragraph 3)
Interpretation	20	Give a cautious overall interpretation of results considering objectives, limitations, multiplicity of analyses, results from similar studies, and other relevant evidence	Discussion (final paragraph)
Generalisability	21	Discuss the generalisability (external validity) of the study results	Discussion (paragraph 3)
**Other information**			
Funding	22	Give the source of funding and the role of the funders for the present study and, if applicable, for the original study on which the present article is based	Acknowledgements section

a Give information separately for exposed and unexposed groups.

Note: An Explanation and Elaboration article discusses each checklist item and gives methodological background and published examples of transparent reporting. The STROBE checklist is best used in conjunction with this article (freely available on the Web sites of PLoS Medicine at http://www.plosmedicine.org/, Annals of Internal Medicine at http://www.annals.org/, and Epidemiology at http://www.epidem.com/). Information on the STROBE Initiative is available at http://www.strobe-statement.org.

References

1. Vandenbroucke, J. P. *et al.* Strengthening the Reporting of Observational Studies in Epidemiology (STROBE): explanation and elaboration. *PLOS Medicine* **4**, e297, DOI: 10.1371/journal.pmed.0040297 (2007).
